# Any heart failure treatments associated with worsening renal function in patients admitted due to acute heart failure?

**DOI:** 10.1080/0886022X.2020.1858100

**Published:** 2021-01-06

**Authors:** Chutatip Limkunakul, Benjawan Srisantithum, Yotin Lerdrattanasakulchai, Thanakorn Laksomya, Jatuphorn Jungpanich, Kittisak Sawanyawisuth

**Affiliations:** a Department of Medicine, Panyananthaphikkhu Chonprathan Medical Center, Srinakharinwirot University, Nonthaburi, Thailand; b Department of Nursing, Panyananthaphikkhu Chonprathan Medical Center, Srinakharinwirot University, Nonthaburi, Thailand; c Department of Medicine, Faculty of Medicine, Khon Kaen University, Khon Kaen, Thailand

**Keywords:** Acute kidney injury, acute renal failure, worsening renal function, diastolic blood pressure, acute heart failure, beta blocker

## Abstract

**Background:**

Worsening renal function (WRF) occurs in approximately 25% of acute heart failure patients, and both baseline characteristics and heart failure treatment may increase the risk of WRF. This study aimed to evaluate additional risk factors for WRF in acute heart failure, particularly those related to heart failure treatment.

**Methods:**

This was a retrospective, observational, analytical study. The inclusion criteria were age 18 years or over, hospital admission due to acute heart failure, and having undergone at least two serum creatinine tests during admission. The eligible patients were classified into two groups: WRF and non-WRF. Predictors for WRF (including treatment parameters) were determined using logistic regression analysis.

**Results:**

During the study period, there were 301 eligible patients who met the study criteria. Of those, 82 (27.24%) had WRF. There were two independent factors associated with WRF occurrence: baseline diastolic blood pressure and beta blocker treatment, with adjusted odds ratios (95% confidence interval) of 1.060 (1.008, 1.114) and 0.064 (0.006, 0.634), respectively. The Hosmer-Lemeshow Chi square for the final model was 6.11 (*p* = .634).

**Conclusions:**

After examining several heart failure treatments and baseline factors, we found that beta blocker treatment results improvement in kidney function.

## Introduction

Acute heart failure is a common cardiac condition that can lead to significant morbidity and mortality and may cause acute kidney injury or worsening renal function (WRF). Previous studies have shown that WRF in acute heart failure can occur as soon as five days after hospital admission [1] and is associated in both short- and long-term morbidity and mortality [2–4]. In-hospital mortality for these patients may be high as 12.93% with a 30-day readmission rate of 41.58% [3]. A recent study also showed that WRF was related to all-cause mortality and cardiovascular mortality at one year after discharge with adjusted hazard ratios of 2.819 and 3.907, respectively [4]. In addition, patients with WRF have been shown to have higher inpatient hospital costs and longer hospital stays than those without (10,977 vs 7,820 USD and 8.2 vs 5.7 days, *p* < .001 for both outcomes) [2].

Approximately 25% of heart failure patients develop WRF (4,734/18,634 patients). Previous studies have examined the risk factors for WRF in acute heart failure [5], which include baseline serum creatinine (odds ratio 3.02), atrial fibrillation (odds ratio 0.35), age over 80 years (odds ratio 2.72), and high systolic blood pressure (odds ratio 1.61) [6,7]. An animal study also showed that renal fibrosis and renal dysfunction may be occurred in ischemic heart disease [8]. Even though there are several heart failure treatment, there are limited data available regarding the effects of heart failure treatment on the risk of WRF. The rationale for this study is that rigorous treatment for heart failure may reduce renal blood flow and cause WRF. We thus aimed to evaluate additional risk factors for WRF in acute heart failure with an emphasis on heart failure treatment.

## Methods

This was a retrospective, observational, analytical study conducted at Panyananthaphikkhu Chonprathan Medical Center at Srinakarinwirot University in Nonthaburi, Thailand. The inclusion criteria were age 18 years or over, hospital admission due to acute heart failure, and having undergone at least two serum creatinine tests during admission. The exclusion criteria were end-stage renal disease with renal replacement therapy, in-hospital heart failure, and having received any treatment for heart failure from other hospitals for a duration of more than 24 h. We included the eligible patients by using the ICD-10 code for acute heart failure: I500, I501, I509, I110, I130, and I132. Admission charts were also reviewed to confirm the diagnosis of acute heart failure according to guideline of the European Society of Cardiology [9]. The study period was from January 2014 to December 2016. The study protocol was approved by the university’s institutional review board (IRB): ID 15.3/2560.

The baseline characteristics, physical signs, laboratory results, and treatment regimens of eligible patients were examined. Treatments were evaluated by the attending physicians. Comorbid diseases, causes of heart failure, severity of heart failure, types of heart failure, and medications were recorded. The primary outcome of the study was WRF, defined as a serum creatinine increase of over 0.3 mg/dL plus percent change of serum creatinine over +25% [7,9].

Sample size calculation. A previous review found that WRF occurred in approximately 25% of patients with acute heart failure [10]. The expected rate of WRF in this study was 33%, resulting in an estimated sample size of 274 patients with a power of 80% and confidence interval of 95% according to a two-sided approach.

Statistical analyses. Eligible patients were classified into either the WRF or non-WRF group. Descriptive statistics were used to compare the differences between the two groups. Predictors for WRF were determined using logistic regression analysis. The unadjusted odds ratios and p values of the studied variables were evaluated using univariate logistic regression analysis. Potentially significant factors according to univariate logistic regression analysis were subjected to subsequent stepwise multivariate logistic regression analysis. The remaining factors in the final model were those with *p* values of less than .25. The goodness of fit of the final model was evaluated using the Hosmer-Lemeshow method. Results are represented as unadjusted/adjusted odds ratios with their 95% confidence intervals. The appropriate cutoff points for numerical WRF predictors were calculated using logistic regression analysis. A receiver operating characteristic curve (ROC) was also created and sensitivities and specificities of the cutoff points reported. The analyses were performed using STATA (College Station, Texas, USA).

## Results

During the study period, there were 301 eligible patients who met the study criteria, 82 (27.24%) of whom had WRF. The median time of presence of WRF was 3 days (range 1-14 days). The only factor that differed significantly between those with and without WRF was history of hypertension. A significantly higher proportion of patients in the WRF group had hypertension as a comorbid disease than in the non-WRF group (84.15% vs 72.60%; p = .049), as shown in Table 1. The physical signs, laboratory results, and treatments that differed significantly between the two groups were fluid therapy, treatment with beta blockers, and B-type natriuretic peptide (BNP) levels (Table 2). The non-WRF group received a greater amount of fluid than those in the WRF group (4030 vs 2656 mL; *p* < .001) and had a greater fluid output. The WRF group received less negative fluid balancing (-442 vs −948 mL; *p* < .001) and had lower rates of beta blocker treatment than the non-WRF group (47.56% vs 62.56%; *p* = .025). The WRF group also had marginally higher baseline serum Creatinine levels and rates of intravenous inotropic use (*p* = .050 and .058). 

**Table 1.** Baseline characteristics of patients admitted with heart failure categorized by presence of worsening renal function (WRF) during hospitalization (*n* = 301).

Factors	No WRF *n* = 219	WRF *n* = 82	*p* Value
Mean (SD) age, year	66.47 (15.69)	67.57 (13.46)	.675
Male sex, *n* (%)	94 (42.92)	27 (32.93)	.146
Mean (SD) Body mass index, kg/m^2^	26.54 (7.15)	27.25 (8.28)	.774
Comorbid diseases, *n* (%)			
Hypertension	159 (72.60)	69 (84.15)	.049
Diabetes	121 (55.25)	50 (60.98)	.433
Coronary artery disease	55 (25.11)	23 (28.05)	.658
Atrial fibrillation	28 (12.79)	12 (14.63)	.704
Dyslipidemia	118 (53.88)	48 (58.54)	.516
Stroke/TIA	16 (7.31)	5 (6.10)	.805
Chronic kidney disease	166 (75.80)	64 (78.05)	.761
Stage I	31 (14.16)	8 (9.76)	.174
Stage II	47 (21.46)	13 (15.85)	
Stage III	58 (26.48)	23 (28.05)	
Stage IV	36 (16.44)	15 (18.29)	
Stage V, no dialysis	9 (4.11)	10 (12.20)	
Unknown	38 (17.35)	13 (15.85)	
NYHA FC, *n* (%)			.222
FC I	3 (1.37)	1 (1.22)	
FC II	8 (3.65)	4 (4.88)	
FC III	105 (47.95)	29 (35.37)	
FC IV	103 (47.03)	48 (58.54)	
Causes of heart failure, *n* (%)			
Acute coronary syndrome	38 (17.35)	21 (25.61)	.141
Hypertensive emergency	24 (10.96)	11 (13.41)	.549
Cardiomyopathy	78 (35.62)	30 (36.59)	.893
Cardiac arrhythmia	34 (15.53)	6 (7.32)	.085

NYHA: New York Heart Association; FC: Functional Classification.

**Table 2.** Physical signs, laboratory results, and treatments in patients admitted with heart failure categorized by presence of worsening renal function (WRF) during hospitalization (*n* = 301).

Factors	No WRF *n* = 219	WRF *n* = 82	*p* Value
Systolic blood pressure, mmHg	150 (34)	156 (39)	.300
Diastolic blood pressure, mmHg	82 (22)	85 (22)	.169
Heart rate, bpm	82 (25)	90 (23)	.437
Oxygen saturation, %	91 (9)	89 (10)	.080
Hemoglobin, g/%	11.3 (2.5)	11.1 (2.3)	.436
Serum Cr, mg/dL	1.58 (1.36)	1.79 (1.34)	.050
Serum sodium, mEq/L	136 (5)	136 (5)	.746
BNP, pg/mL	9212 (10160)	17646 (26402)	.037
Ejection fraction, %	47 (17)	47 (18)	.739
Ejection fraction ≤40, *n* (%)	80 (36.53)	36 (43.90)	.287
Net balance/day, ml	−948.45 (1262.15)	−442.49 (973.12)	<.001
Intravenous furosemide, mg	183 (472)	397 (1117)	.078
Oral furosemide, mg	76 (183)	68 (169)	.563
Total dose of furosemide, mg	259 (561)	465 (1144)	.436
Intravenous inotropic agent, *n* (%)	18 (8.22)	13 (15.85)	.058
Intravenous nitroglycerin, *n* (%)	33 (15.07)	13 (15.85)	.859
Thiazide use, *n* (%)	4 (1.83)	0	.578
Dose, mg	34.37 (11.96)	0	NA
Spironolactone, *n* (%)	12 (5.48)	3 (3.66)	.767
Dose, mg	71.67 (77.10)	91.66 (94.64)	.884
ACEI/ARB, *n* (%)	89 (38.81)	28 (34.15)	.505
CCB, *n* (%)	69 (31.51)	23 (28.05)	.673
Betablocker, *n* (%)	137 (62.56)	39 (47.56)	.025
Nephrotoxic agent, *n* (%)			
Contrast media	3 (1.37)	0	.565
Antibiotic	6 (2.74)	2 (2.44)	.999
NSAIDs	1 (0.46)	0	.999

Data presented as mean (SD) unless indicated otherwise; BNP: B-type natriuretic peptide; ACEI: angiotensin converting enzyme inhibitors; ARB: angiotensin receptor blocker; CCB: calcium channel blocker; NYHA: New York Heart Association; FC: Functional Classification; fluid therapy included both oral and intravenous forms.

The final model for predicting the WRF, in which two factors remained, is shown in Table 3. There were two independent factors for WRF occurrence: baseline diastolic blood pressure and beta blocker treatment (bold, Table 3) with adjusted odds ratios (95% confidence interval) of 1.060 (1.008, 1.114) and 0.064 (0.006, 0.634), respectively. The Hosmer-Lemeshow Chi square for the final model was 6.11 (*p* = .634). Diastolic blood pressure of 70 mmHg had a sensitivity of 80.49% and specificity of 32.42% for WRF, while the cutoff point of 100 mmHg resulted in a sensitivity of 25.61% and specificity of 79.45%. The area under the ROC curve for diastolic blood pressure was 55.15%.

**Table 3.** Factors associated with worsening renal function during hospitalization in patients admitted with heart failure.

Factors	Unadjusted odds ratio (95% confidence interval)	Adjusted odds ratio (95% confidence interval)
Diastolic blood pressure	1.007 (0.996, 1.018)	1.060 (1.008, 1.114)
Beta blocker treatment	0.542 (0.325, 0.906)	0.064 (0.006, 0.634)

## Discussion

Of the several treatments categorized as class I recommendations in the ACC heart failure guidelines, we found that and beta blockers reduced WRF by 94% (Table 3). Previous studies have found beta blockers to be associated with reductions in mortality of 20-50%, regardless of GFR, EF, or sinus rhythm [11–14]. The beneficial effects of beta blockers in cases of heart failure may last for up to one year [15]. Preserved EF heart failure patients with heart rates of 60 beats/minute or lower have better mortality outcomes [16]. One possible mechanism to explain the favorable effects of beta blockers in heart failure is that they increase renal tissue oxygenation by lowering renal oxygen consumption [17]. A previous study also found that beta blockers decrease vascular resistance by 16%, increasing glomerular filtration rates and renal plasma flow by 10% and 13%, respectively, which may also improve renal function [18].

This study also found that presenting diastolic blood pressure was associated with WRF. Although two previous studies found that systolic blood pressure (either less than 90 or over 160 mmHg) was significantly associated with WRF [7,19], neither evaluated the effects of diastolic blood pressure. In our study, only diastolic blood pressure, and not systolic blood pressure, was independently related to WRF, with a 6% increase in risk of WRF per 1 mmHg increase in diastolic blood pressure.

Several studies have shown high diastolic blood pressure to be associated with declines in renal function. One study conducted on 86 patients with renal insufficiency found that diastolic blood pressure lower than 90 mmHg was associated with a slower progression of end stage renal disease but that systolic blood pressure was not related [20]. High diastolic blood pressure may also indirectly indicate nephrosclerosis. A previous study found that 64% of patients with nephrosclerosis had diastolic blood pressure over 90 mmHg [21]. However, the mechanisms by which high diastolic blood pressure leads to deterioration of renal function, especially in acute cases, requires further study.

There are some limitations to this study. First, treatments provided were done so at the complete discretion of the attending physicians. No restrictive study protocol was applied. Second, there were no data regarding beta blocker treatment pre-hospitalization including dosage or type. Third, the WRF was found lower than expected rate in sample size calculation (27.24% vs 33%). This finding may result in lower statistical power. Fourth, some related factors were not studied [22–24]. However, there was a significant predictor from the analysis. Finally, there were no long-term follow-up data or time to develop WRF. Due to retrospective study design, the time to develop WRF in this study was approximately 3 days.

## Conclusion

After examining several heart failure treatments and baseline factors, we found that beta blocker treatment results improvement in kidney function.
